# Independent and Confluent Middle Mesial Root Canals in Mandibular First Molars: A Report of Four Cases

**DOI:** 10.1155/2012/103125

**Published:** 2012-06-25

**Authors:** Mohanavelu Deepalakshmi, Chellasamy Savarimala Karumaran, Revathi Miglani, Rajamani Indira

**Affiliations:** ^1^Department of Conservative Dentistry & Endodontics, Chettinad dental college, Chettinad Health City & Research Institute, Old Mahabalipuram Road, Kelambakkam 603103, Chennai, India; ^2^Department of Conservative Dentistry & Endodontics, Ragas Dental College & Hospital, Uthandi 600119, Chennai, India

## Abstract

Mandibular molars demonstrate considerable variations with respect to number of roots and root canals. The possibility of additional root canals should be considered even in teeth with a low frequency of abnormal root canal anatomy. This paper discusses the endodontic management of the rare anatomical complexity middle mesial canals in mandibular first molar and also serves to remind the clinicians that such anatomical variations should be taken into account during the endodontic treatment of the mandibular molars.

## 1. Introduction

Knowledge of both normal and abnormal anatomy of the root canal system dictates the parameters for execution of root canal therapy and can directly affect the outcome of the endodontic therapy. Missed extra roots and root canals are a major reason for failure of root canal treatment [1]. All categories of teeth may have additional roots and/or canals, with an increased likelihood of finding aberrant canal configurations in premolars and molars [2]. Lower mandibular molars are the first permanent teeth to erupt and most often require endodontic treatment [3]. The lower mandibular first molar normally has two roots, one mesial and one distal with two canals in the mesial and one or two canals in the distal root. The literature cites the anatomic variations and abnormalities associated with lower first mandibular molars; variations in canals include C-shaped canals, five canals, six canals, and seven canals. Variations in roots like three rooted mandibular molars have also been reported [2, 4–9]. 

Till date few clinical reports have described more than two canals in the mesial root of mandibular molars. Among these, the occurrence of middle mesial canal in the lower mandibular molar is (1–15%); this canal is also called “intermediary mesial canal” or “medial mesial canal” since it is situated centrally between the main buccal and lingual root canals [10–17]. The diameter of these middle mesial canals is smaller than other two [10] and is age related due to dentinal apposition [5]. The mesial canal is called independent when a distinct coronal orifice and apical foramen were observed or confluent when converging to one of the other two main canals and terminating at a common foramen [4].

It is of prime importance for the clinician to identify the entire topographic location of any additional canal orifices and also extremely important that clinicians use all the armamentaria at their disposal to locate and treat the entire root canal system [18]. Well-angulated periapical films should be taken with cone-directed straight-on, mesio-oblique, and disto-oblique; this technique often reveals and clarifies the three dimensional morphology of the tooth. The use of the magnifying loupes, dental operating microscope, and adjunctive diagnostic aids like cone beam CT and so forth can also be used [19, 20]. 

This paper reviews the endodontic management of 4 cases, of a mandibular first molar with three mesial canals (independent and confluent) in the mesial root, where 2 cases were managed under magnifying loupes and 2 cases under dental operating microscope.

## 2. Case Report 

### 2.1. Case  1

**Figure 1:**
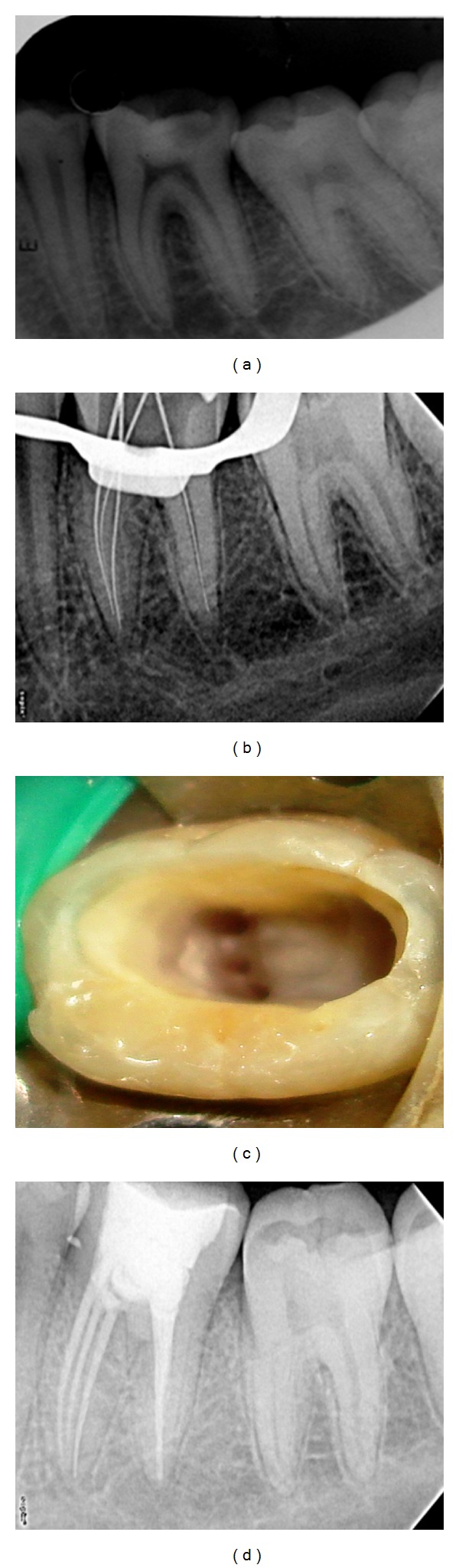
(a) Preoperative radiograph. (b) Working length. (c) Clinical image. (d) Obturation.

A 19-year-old female patient was referred to the Department of Endodontics, Ragas Dental College and Hospital, with the chief complaint of intermittent pain in the lower right back teeth for the past three months. Her past medical history was found to be noncontributory. Clinical examination revealed a deep carious lesion in right mandibular first molar no. 46. The clinical and radiographic findings led to a diagnosis of chronic irreversible pulpitis of the right mandibular first molar no. 46, necessitating endodontic therapy. Radiographic evaluation of the involved tooth indicated a deep, carious lesion approximating the pulp and a normal canal configuration of two canals in the mesial root and one canal in the distal root (Figure 1(a)). The right inferior alveolar nerve was anesthetized using 2% Lignocaine with 1 : 80,000 adrenaline (Lignox, Indoco Remedies Ltd, India). The tooth was isolated using rubber dam, and an endodontic access cavity was established. Investigation of the root canal system was initially performed with the aid of an endodontic explorer, and the canals were explored with a no. 10 K-file (Mani, Inc; Tochigi, Japan), two canals mesially and single canal distally were located initially. In order to enhance the visualisation, we observed the pulp chamber with the aid of an magnifying loupes (Seiler loupes, 2.5× magnification). Examining the fissure connecting the two mesial canals revealed an additional orifice in between the two mesial canals (mesiobuccal, middle mesial, and mesiolingual). The additional canal was explored with a no. 10 K-file (Mani, Inc; Tochigi, Japan). 

Multiple working length radiographs were taken at different angulations with one file placed in each of the 3 mesial and single distal orifice to confirm the independent presence of 4 distinct canals (Figure 1(b)). Individual canal instrumentation was performed using a crown down preparation with Protaper nickel-titanium rotary instruments (Maillefer, Dentsply, Ballaigues, Switzerland). Copious chemical irrigation was performed with 5.25% sodium hypochlorite solution and EDTA (Glyde, Maillefer, Dentsply, Ballaigues, Switzerland). The root canals were dried with paper points (Maillefer, Dentsply, Ballaigues, Switzerland), and the pulp chamber was examined again under the magnifying loupes (Seiler loupes, 2.5× magnification) (Figure 1(d)). Obturation was performed after two weeks with resin sealer (AH plus-Dentsply, DeTrey Konstanz, Germany) and cold laterally condensed with gutta-percha (Mailllefer, Dentsply, Tulsa, OK) and sealed with IRM cement. Postobturation radiograph was taken to confirm the completeness and the extension of the root filling, which revealed three distinct orifices with three separate apical terminations of mesial canals with thin dentinal separation between three canals till apical third. With regard to Pomeranz's classification this case is classified as independent middle mesial canal and type VIII root canal morphology according to Vertucci's classification (Figure 1(c)).

### 2.2. Case  2

**Figure 2:**
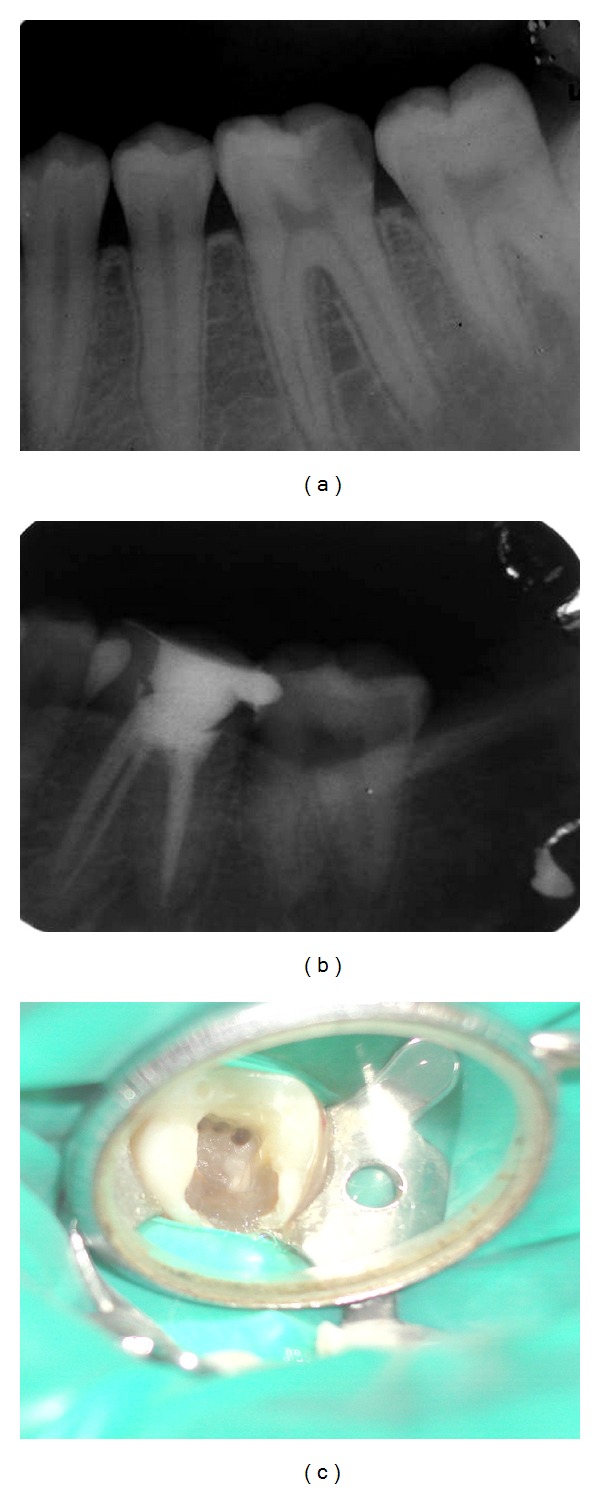
(a) Preoperative radiograph. (b) Obturation. (c) Clinical image.

A 35-year-old female patient reported with spontaneous pain in lower right back tooth indicative of chronic irreversible pulpitis no. 46 (Figure 2(a)). After anesthesia and rubber dam placement, access cavity was prepared and the coronal pulp tissue was removed. Totally 5 distinct orifices-3 located mesially (mesiobuccal, middle mesial and mesiolingual) and 2 distally (distobuccal and distolingual) (Figure 2(c)). The canals were explored with no. 10 K-file (Mani, Inc; Tochigi, Japan). The working length radiograph confirmed the presence of 5 distinct orifices and 4 apical terminations. Chemomechanical preparation was performed using the step back technique and the root canals were obturated with cold, laterally condensed gutta-percha (Maillefer, Dentsply, Ballaigues, Switzerland), and resin sealer (AH plus sealer-Maillefer, Dentsply, Ballaigues, Switzerland). Postobturation radiograph (Figure 2(b)) revealed the presence of confluent middle mesial canal originated as a separate orifice but joined in the apical third of the mesiobuccal canal.

### 2.3. Case  3

**Figure 3:**
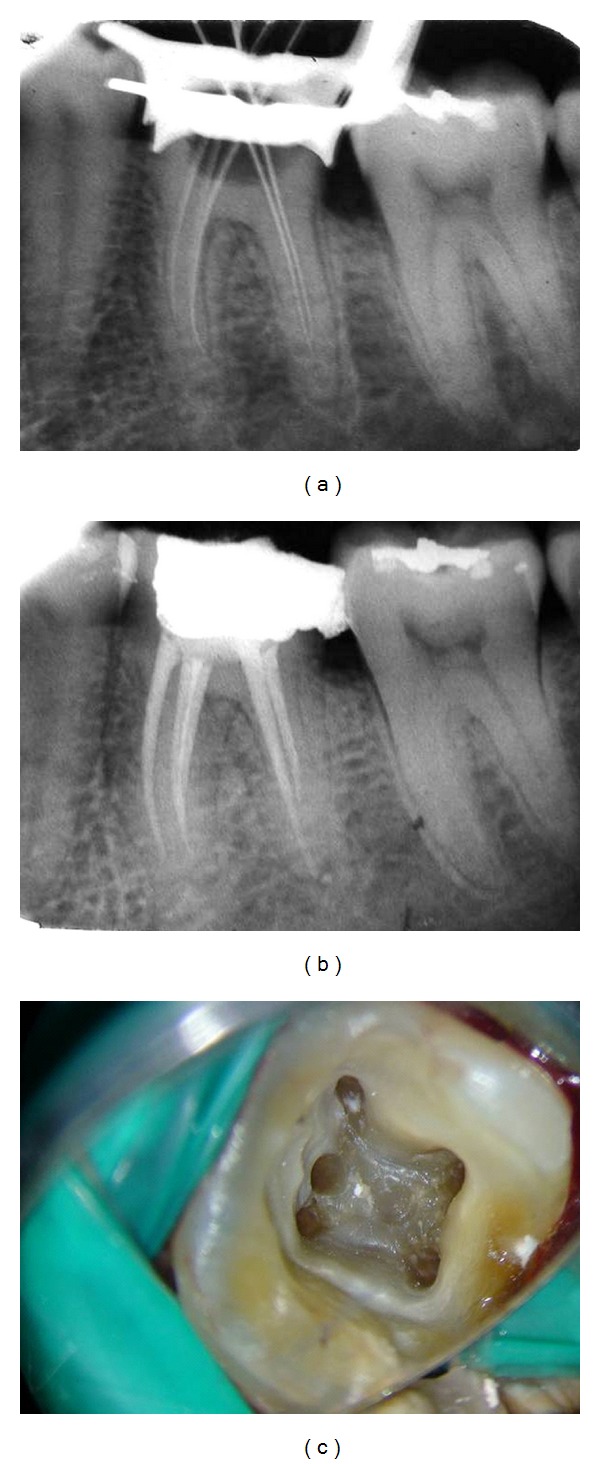
(a) Working length. (b) Obturation. (c) Clinical image.

A 28-year-old female patient reported with spontaneous pain in lower right back tooth no. 46. Endodontic treatment was indicated because of apical periodontitis. After anesthesia and rubber dam placement, access cavity was prepared and pulp tissue was removed. Totally 5 distinct orifices-3 located mesially (mesiobuccal, middle mesial, and mesiolingual) and 2 distally (distobuccal and distolingual) (Figure 3(c)) were detected on inspection using operating microscope. The working length radiograph confirmed the presence of 5 distinct orifices, and 4 apical terminations confluent middle mesial canal originated as a separate orifice but joined in the apical third of the mesio buccal canal (Figure 3(a)). Chemomechanical preparation was performed using the step back technique and obturated with cold, laterally condensed gutta-percha (Maillefer, Dentsply, Ballaigues, Switzerland) and resin sealer (AH plus -Maillefer, Dentsply, Ballaigues, Switzerland). Postobturation radiograph revealed confluent middle mesial canal and (additional type 3-2) (Figure 3(b)).

### 2.4. Case  4

**Figure 4:**
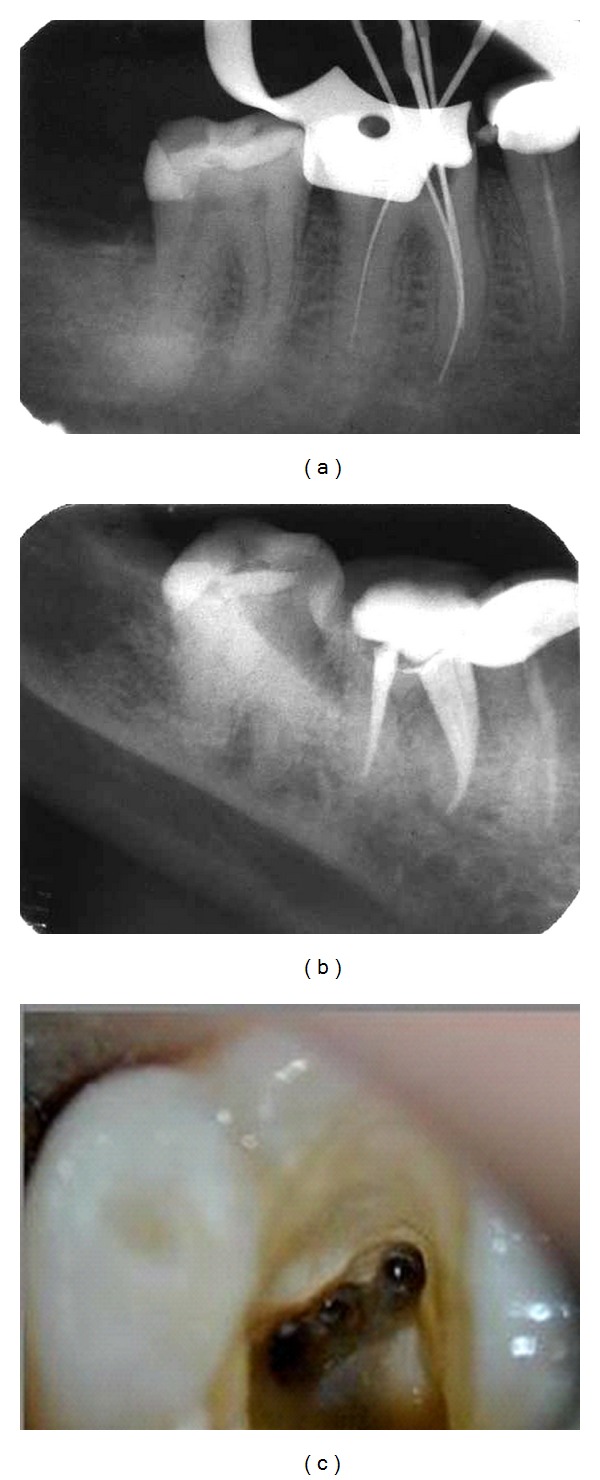
(a) Working length. (b) Obturation. (c) Clinical image.

A 24-year-old female patient was referred for root canal treatment of lower left mandibular first molar owing to irreversible pulpitis no. 36. Access preparation was performed and pulp tissue extirpated under local anesthesia. One distal and 2 mesial canal orifices were revealed on inspection using operating microscope under 12.8× magnifications (Figure 4(c)). After probing with a Hu-Friedy (Chicago, IL) DG 16 endodontic explorer, a small hemorrhagic point was noticed in between two mesial canal orifices. A working length radiograph was taken that confirmed the presence of 4 canals (Figure 4(a)). Cleaning and shaping were done and filled with cold, laterally condensed gutta-percha and AH plus resin sealer. Postobturation radiograph (Figure 4(b)) reveals 1 distal (vertucci type 1) 3 mesial canals where the middle mesial canal is joined with mesiobuccal canal (confluent), (additional types 3-2).

## 3. Discussion

The present paper reports the endodontic management of independent and confluent middle mesial canals in the mandibular first molars. Unusual canal anatomy associated with the mandibular first molar has been reported in several studies. Fabra et al. [5] reported that 2.6% of molars had three canals in the mesial root, 1.7% of third canal joined the mesiobuccal canal in the apical third, and 1.6% converged with the mesiolingual canal and as an independent canal (0.13%). Goel et al. [9] reported that mandibular first molars had 13.3% three mesial canals, 3.3% four mesial canals, and 1.7% three distal canals. They also reported that one apical foramen was present in 30%, two in 60%, three in 6.7%, and four in 3.3% of the cases. The occurrence of three independent canals in the mesial root similar to first case in this paper is most uncommon manifestation and rarely encountered [2, 5, 13, 14]. 

The mesial roots of mandibular first and second molars had mostly one large canal until 11 and 15 years of age; due to secondary dentine depositions at 30–40 years of age, the canal system in the apical and middle third of the root was completely established. There are also chances of extensive differentiation resulting in reticular form. The prevalence of intercanal communications were low at young and old ages, but high at intermediate ages. It is important to be familiar with these age-related variations in the root canal system to aid in the location and negotiation of canals as well as their subsequent management [17, 21].

 Prevention of missed anatomy starts with good preoperative radiographs, even though radiographs have limitations in assessing the number of canals radiographs taken from at least two different horizontal angles along with careful interpretation, which will aid in the detection of extra canals [22]. Without doubt, a proper access cavity preparation is of central importance in localizing the orifices of the root canals, examination of the pulp chamber floor with a sharp explorer, troughing of grooves with ultrasonic tips, staining the chamber floor with 1% methylene blue dye, and performing the sodium hypochlorite “champagne bubble” test, fibrooptic transillumination, and visualizing canal bleeding points are important aids in locating root canal orifices. Clinically, the presence of additional canal indicates continuous bleeding in teeth with pulpitis or normal pulps despite complete instrumentation; Clinically, the presence of additional canal indicates continuous bleeding in teeth with pulpitis or normal pulps despite complete instrumentation. Following are the important aids in locating the additional canal such as: the presence of apical rarefaction on the lateral side of the root with necrotic pulps, fast break guideline, eccentric location of an endodontic file during working length determination, inconsistent apex locator readings, a sinus tract that traces laterally away from the main canal, or the feeling of a “catch” on the canal wall during instrumentation [23].

In most of the cases, middle mesial canal is hidden by a dentinal projection in the mesial aspect of pulp chamber walls, and this dentinal growth is usually located between the two main canals (mesiobuccal-mesiolingual). Ultrasonic systems provide a breakthrough for exploring and identifying the extra canals and also eliminate the bulky head of the conventional hand piece that frequently obstructs the vision. The working tips of the specific ultrasonic instruments are ten times smaller than the smallest round bur, and their abrasive coating aids in a controlled and delicate removal of calcifications and other interferences of the canal orifices [18]. Dental loupes are one of the most common magnification system used in dentistry [24]. In this paper, the first two cases were managed endodontically using dental loupes. The use of dental operating microscope provides enhanced lighting and visibility and identifies subtle color changes, better understanding of floor map, fine instrumentation, coaxial illumination, and magnification. In the present paper, the middle mesial canal was present at an equidistance between the two mesial canals (mesiobuccal and mesiolingual).

Failure to recognise the anatomy of a root canal system and developmental anomalies might lead to inadequate debridement of the root canal system and thus contribute to unfavourable endodontic treatment outcome and the subsequent need for retreatment or surgical intervention [18]. The present paper necessitates for thorough investigation of root canal anatomy during treatment to clean and shape it more efficiently. 

## 4. Conclusion

To summarize, treating additional canals may be challenging, but the inability to find and properly treat the root canals may cause failures. Information on root canal anatomy come from radiographs is valuable and should always be integrated with a careful clinical examination, preferably under magnification for the better and successful endodontic treatment.
